# Seroconversion following the first, second, and third dose of SARS-CoV-2 vaccines in immunocompromised population: a systematic review and meta-analysis

**DOI:** 10.1186/s12985-022-01858-3

**Published:** 2022-08-08

**Authors:** Mohammad-Mehdi Mehrabi Nejad, Parnian Shobeiri, Hojat Dehghanbanadaki, Mohammadreza Tabary, Armin Aryannejad, Abdolkarim Haji Ghadery, Mahya Shabani, Fatemeh Moosaie, SeyedAhmad SeyedAlinaghi, Nima Rezaei

**Affiliations:** 1 Department of Radiology, School of Medicine, Advanced Diagnostic and Interventional Radiology Research Center (ADIR), Imam Khomeini Hospital, Tehran University of Medical Sciences (TUMS), Tehran, Iran; 2 School of Medicine, Tehran University of Medical Sciences, Tehran, Iran; 3 Non–Communicable Diseases Research Center, Endocrinology and Metabolism Population Sciences Institute, Tehran University of Medical Sciences, Tehran, Iran; 4 Department of Immunology, Research Center for Immunodeficiencies, Pediatrics Center of Excellence, Children’s Medical Center, Tehran University of Medical Sciences, Qarib St, Keshavarz Blvd, Tehran, 1419733141 Iran; 5 Network of Immunity in Infection, Malignancy and Autoimmunity (NIIMA), Universal Scientific Education and Research Network (USERN), Tehran, Iran; 6 Division of Pulmonary, Allergy and Critical Care Medicine, Department of Medicine, University of Pittsburgh, Pittsburgh, PA USA; 7 Experimental Medicine Research Center, Tehran University of Medical Sciences, Tehran, Iran; 8 Iranian Research Center for HIV/AIDS, Iranian Institute for Reduction of High-Risk Behaviors, Tehran University of Medical Sciences, Imam Khomeini Hospital Complex, Keshavarz Blvd., Tehran, 1419733141 Iran

**Keywords:** COVID-19, SARS-CoV-2, Vaccination, Immunocompromised patient, Malignancy, Transplantation, Autoimmune, Efficacy

## Abstract

**Background:**

Immunocompromised (IC) patients are at higher risk of more severe COVID-19 infections than the general population. Special considerations should be dedicated to such patients. We aimed to investigate the efficacy of COVID-19 vaccines based on the vaccine type and etiology as well as the necessity of booster dose in this high-risk population.

**Materials and methods:**

We searched PubMed, Web of Science, and Scopus databases for observational studies published between June 1st, 2020, and September 1st, 2021, which investigated the seroconversion after COVID-19 vaccine administration in adult patients with IC conditions. For investigation of sources of heterogeneity, subgroup analysis and sensitivity analysis were conducted. Statistical analysis was performed using R software.

**Results:**

According to the Preferred Reporting Items for Systematic Reviews and Meta-Analyses, we included 81 articles in the meta-analysis. The overall crude prevalence of seroconversion after the first (n: 7460), second (n: 13,181), and third (n: 909, all population were transplant patients with mRNA vaccine administration) dose administration was 26.17% (95% CI 19.01%, 33.99%, I^2^ = 97.1%), 57.11% (95% CI: 49.22%, 64.83%, I^2^ = 98.4%), and 48.65% (95% CI: 34.63%, 62.79%, I^2^ = 94.4%). Despite the relatively same immunogenicity of mRNA and vector-based vaccines after the first dose, the mRNA vaccines induced higher immunity after the second dose. Regarding the etiologic factor, transplant patients were less likely to develop immunity after both first and second dose rather than patients with malignancy (17.0% vs 37.0% after first dose, *P* = 0.02; 38.3% vs 72.1% after second dose, *P *< 0.001) or autoimmune disease (17.0% vs 36.4%, *P* = 0.04; 38.3% vs 80.2%, *P *< 0.001). To evaluate the efficacy of the third dose, we observed an increasing trend in transplant patients after the first (17.0%), second (38.3%), and third (48.6%) dose.

**Conclusion:**

The rising pattern of seroconversion after boosting tends to be promising. In this case, more attention should be devoted to transplant patients who possess the lowest response rate.

**Supplementary Information:**

The online version contains supplementary material available at 10.1186/s12985-022-01858-3.

## Introduction

Severe acute respiratory syndrome coronavirus 2 (SARS-CoV-2) was firstly reported in Wuhan, Hubei Province, China, in December 2019 [1, 2]. Due to the rapid global spread of SARS-CoV-2, leading to thousands of deaths by the coronavirus disease (COVID-19), the World Health Organization (WHO) declared a pandemic on March 12th, 2020. COVID-19 has put a massive burden on the world in the case of human lives lost, economic consequences, and increasing poverty over the last two years [3]. From the first waves of the pandemic, researchers have struggled to develop an effective and safe vaccine against this virus, and some were developed and passed the trial phase expeditiously [4].

Some vaccines have been approved by the WHO so far, including messenger RNA (mRNA) vaccines, including mRNA-1273 Moderna and BNT162b2 Pfizer BioNTech, viral vector vaccines, namely AstraZeneca and Janssen Ad26.COV2.S, and inactivated virus vaccines, including Sinovac and Sinopharm [5]. Concerning immunogenicity and safety of these vaccines, preliminary reports from phase II/III and some real-world data are available to date [6–9]; however, little attention has been paid to immunocompromised (IC) patients since such patients were not included in the primary trials of the above-mentioned vaccines [10]. IC patients, including those with primary immunodeficiencies, autoimmune diseases, malignancies, human immunodeficiency virus (HIV) infection, and those taking immunosuppressive agents, are at higher risk of more severe SARS-CoV-2 infections than the general population [11–15]. So, special considerations should be dedicated to such patients, and investigating the efficacy and safety of vaccines against SARS-CoV-2 is crucial in these patients.

Heterogeneous studies have recently assessed the immune response against SARS-CoV-2 in IC patients after receiving the first, second, or the third dose of approved vaccines, mostly by assessing the SARS-CoV-2 anti-spike or anti-receptor-binding domain (RBD) antibodies [16–18]. In this systematic review and meta-analysis, we aimed to provide a more explicit vision by systematically reviewing the literature and complementing the reported clinical outcomes around the efficacy of vaccines in IC patients.

## Methods

Seroconversion frequencies following vaccination were studied using the Preferred Reporting Items for Systematic Reviews and Meta-Analyses (PRISMA) framework [19] and a systematic search to locate relevant research papers.

### Search strategy and databases

PubMed-MEDLINE, Scopus, and Web of Science were searched for original articles reporting the seroconversion after COVID-19 vaccine administration in adult patients with IC conditions between June 1st, 2020, and September 1st, 2021. The search terms were as follows: ((COVID-19) OR (SARS-CoV-2) OR (novel coronavirus)) AND ((vaccine) OR (vaccination) OR (vaccinated)) AND ((immunocompromised) OR (immunosuppressed) OR (corticosteroid) OR (chemotherapy) OR (cancer) OR (malignancy) OR (rheumatologic disease) OR (immunodeficiency) OR (autoimmune) OR (AIDS) OR (HIV) OR (transplant)).

### Selection criteria

Studies examining the prevalence of seroconversion following COVID-19 immunization in IC patients met the inclusion criteria. The papers considered in this review satisfied the following criteria: (1) Population: studies including ≥ 30 IC patients. IC patients included those receiving chemotherapy for solid organ or hematologic malignancies, those with hereditary or acquired immunodeficiency illnesses, those with autoimmune or rheumatologic diseases, and those with other ailments (e.g., asthma) getting long-term corticosteroid treatment. (2) Intervention: immunization against COVID-19 (3) Outcomes: The primary outcome measure in this study was seroconversion in IC patients who had anti-SARS-CoV-2 spike IgG ≥ 14 days after receiving the first, second, and third doses of COVID-19 vaccinations. (4) Design of the study: we included all retrospective and prospective observational studies. The following articles were excluded from consideration: (1) reviews and editorials; (2) case reports or case series including < 30 patients; (3) partially overlapping patient cohorts; (4) non-English literature; and (5) non-human experiments. Two reviewers separately conducted a consensual evaluation of the literature.

### Data extraction

Two experts independently assessed eligible studies and retrieved the following data from each included publication: author, publication date, country of origin, study design, study sample size, the definition of IC conditions, inclusion and exclusion criteria, number of IC patients, variables matched, male/female ratio, mean age, duration of disease, type and etiology of immunodeficiency and its proportion in the total population, and the type of vaccine. Any discrepancies in data extraction were handled by discussion or consultation with a third expert.

### Quality assessment

We evaluated the included studies using the National Institutes of Health (NIH) quality assessment tool [20]. If an element of the criteria was inadequately addressed, not applicable, or not reported in a study, and it could not be identified indirectly, we did not allocate a score to that element. For cohort and cross-sectional studies, 11–14 was considered good, 6–10 fair, and 0–5 poor. The corresponding values were 7–9, 4–6, and 0–3 for the case series and 9–12, 5–8, and 0–4 for case-control studies, respectively.

### Statistical analysis

We used the 'metaprop' function to estimate Der Simonian and Laird's pooled effect on the prevalence of seroconversion following vaccine delivery using a random-effect model. A forest plot was created to depict the summary of meta-analysis findings and heterogeneity. A funnel plot was used to check for publication bias, and Egger's regression tests were used to test for it more objectively, with a *p *< 0.05 deemed to suggest possible publication bias. The Cochrane Q statistic was used to assess between-study heterogeneity [21]. I^2^ was used to assess between-study heterogeneity, with values of 0, 25, 50, and 75% representing no, low, medium, and substantial heterogeneity, respectively [22]. A leave-one-out sensitivity analysis was used to determine the impact of a single study on the total meta-analysis estimate (Additional file 1: Figs. S1-3). The final results were given as text, tables, and figures. All computations and visualizations were carried out using R version 4.0.4 (R Core Team [2020]. R: A language and environment for statistical computing. R Foundation for Statistical Computing, Vienna, Austria), and STATA 16 (StataCorp. 2019. Stata Statistical Software: Release 16. College Station, TX: StataCorp LLC) for Egger’s plots. We used following packages: “meta” (version 4.17-0), “metafor” (version 2.4-0), “dmetar” (version 0.0-9), and “tidyverse” (version 1.3.0). All forest plots, funnel plots, and the drapery plot were designed using R. A *p *< 0.05 was considered statistically significant.

## Results

### Selection of studies

**Fig. 1:**
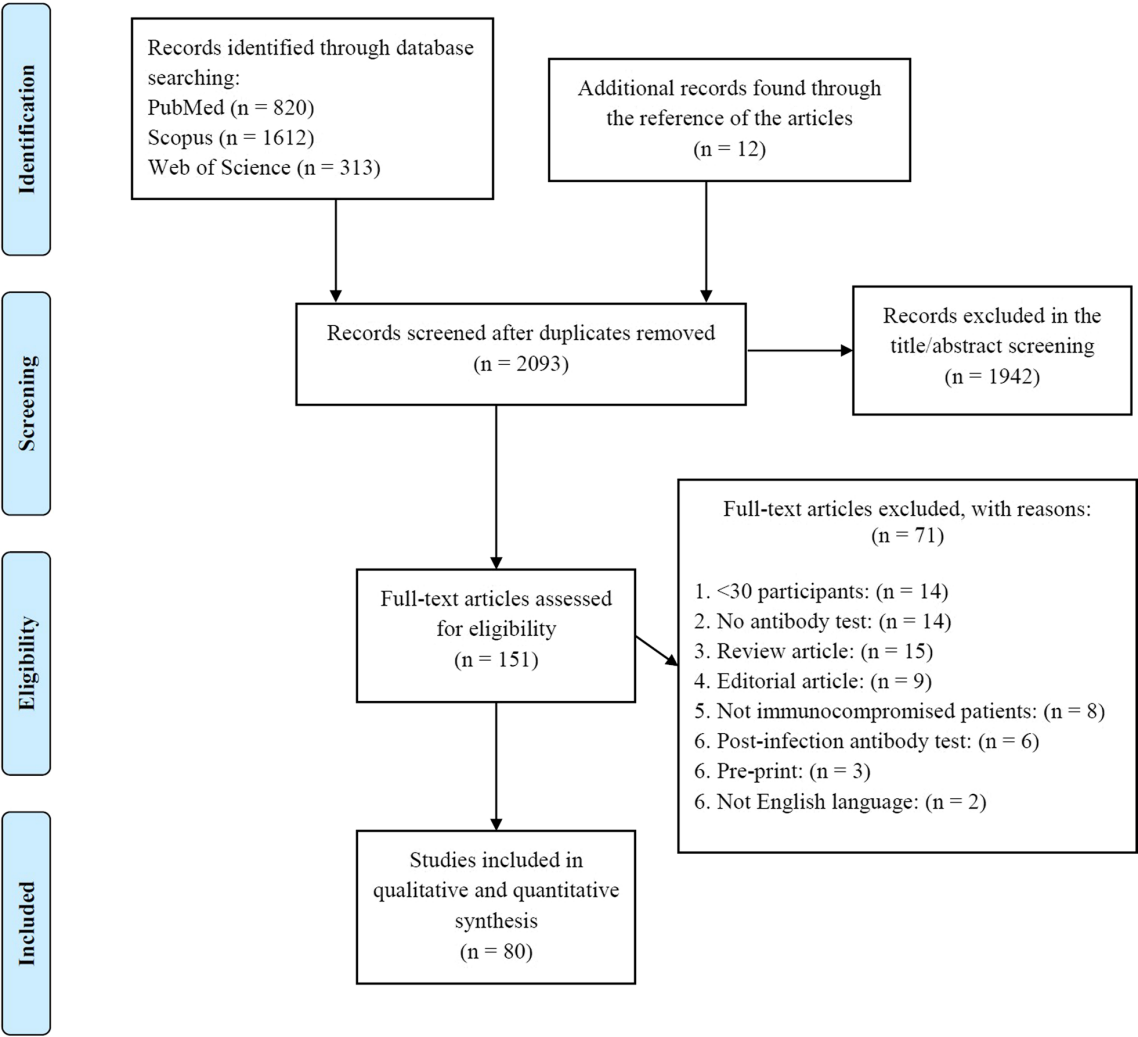
Study selection process according to the preferred reporting items for systematic reviews and meta-analyses (PRISMA) guideline

After implementing our strategy, we reached a total of 2093 research publications. Then, we screened both the titles and abstracts for relevant studies and 151 research articles were selected for full-text screening. Ultimately, 80 research publications [23–102] were included in our systematic review and meta-analyses (Fig. 1; PRISMA diagram).

### Study characteristics

Table 1 summarizes the characteristics of the 80 included studies, which were published in 2021. Forty [23–62] studies assessed seroconversion in immunocompromised patients after the administration of the first dose of the vaccines. Also, 64 [23–25, 28, 30, 33–40, 42, 43, 45–50, 52, 60–101] studies were included as they evaluated seroconversion after the second injection in immunocompromised patients. Lastly, seven [28, 38, 61, 87, 97, 100, 102] studies investigated seroconversion and its prevalence after the third dose of the vaccines. Considering the type of the administered vaccine, we grouped the included studies as mRNA, vector, and inactivated virus. Moreover, regarding the etiology, studies were grouped into autoimmune, malignancy, and transplant.

**Table 1:** Details of the data presented by the included studies

Study (first author)	Country	Study design	Total sample size	Case group			Etiology of IC condition	Type of vaccine
				No. of cases	Male%	Age (mean ± SD) (median [IQR]*)		
Addeo, A.	Switzerland and USA	Prospective cohort	131	131	55	63 [55–69]*	Malignancy	n = 30 (BNT162b2 (Pfizer/BionTech)) or n = 93 (mRNA-1273 (Moderna))
Agbarya, A.	Israel	Cross-sectional	355	140	54	65.3 ± 1.4	Malignancy	BNT162b2 (Pfizer/BionTech)
Agha, M.	USA	Prospective cohort	67	67	52.2	71 [65–77]*	Malignancy	BNT162b2 (Pfizer/BionTech)
Ammitzbøll, C.	Denmark	Retrospective cohort	134	134	67.1	NA	Autoimmune	BNT162b2 (Pfizer/BionTech)
Benotmane, I.	France	Cross-sectional	241	241	64.7	57.7 [49.3-67.6]*	Transplant	mRNA-1273 (Moderna)
Benotmane, I.	France	Prospective cohort	159	159	61.6	57.6 [49.6-66.1]*	Transplant	mRNA-1273 (Moderna)
Bertrand, D.	France	Retrospective cohort	55	45	51	63.5±16.3	Autoimmune	BNT162b2 (Pfizer/BionTech)
Boekel, L.	Netherlands	Prospective cohort	921	632	33	63±11	Autoimmune	ChAdOx1 nCoV-19 (AstraZeneca), BNT162b2 (Pfizer-BioNtech), CX-024414 (elasomeran; Moderna), and Ad.26.COV2.S (Janssen)
Boyarsky, B.	USA	Prospective cohort	1040	1012	NA	60.0 [45.7-68.1]*	Transplant	BNT162b2 (Pfizer/BionTech)
Boyarsky, B.	USA	Prospective cohort	436	423	39	55.9 [41.3-67.4]*	Transplant	n = 223 (BNT162b2 (Pfizer/BionTech)) or n = 204 (mRNA-1273 (Moderna))
Boyarsky, B.	USA	Prospective cohort	123	123	5	50 [41–61]*	Autoimmune	n = 64 (BNT162b2 (Pfizer/BionTech)) or n = 59 (mRNA-1273 (Moderna))
Boyarsky, B.	USA	Prospective cohort	658	658	50	NA	Transplant	n =100 (BNT162b2 (Pfizer/BionTech)) or n = 99 (mRNA-1273 (Moderna))
Boyarsky, B.	USA	Prospective cohort	737	737	42	56 [42–60]*	Transplant	n = 12 (Ad26 (JANSSEN/JOHNSON&JOHNSON)) or n = 725 (mRNA vaccine)
Braun-Moscovici, Y.	Israel	Prospective cohort	290	264	24	57.6 ± 13.18	Autoimmune	BNT162b2 (Pfizer/BionTech)
Cao, J.	USA	Retrospective cohort	47	37	72.9	64 [50–69]*	Transplant	BNT162b2 (Pfizer/BionTech) or mRNA-1273 (Moderna)
Chavarot, N.	France	Retrospective cohort	97	97	58	63.5 [51–72]*	Transplant	BNT162b2 (Pfizer/BionTech)
Chavarot, N.	France	Retrospective cohort	101	101	67.3	64 [53–73]*	Transplant	BNT162b2 (Pfizer/BionTech)
Chevallier, P.	France	Prospective cohort	138	112	59.8	57 [20–75]*	Transplant	BNT162b2 (Pfizer/BionTech)
Chiang, T. P.	USA	Prospective cohort	1039	1039	6.1	NA	Autoimmune	n = 45 (Ad26 (JANSSEN/JOHNSON&JOHNSON)) or n = 994 (mRNA vaccine)
Cohen, D.	Israel	Prospective cohort	137	137	54.7	68.5	Malignancy	BNT162b2 (Pfizer/BionTech)
Cucchiari, D.	Spain	Prospective cohort	148	117	67.3	59.0 ± 52.4	Transplant	mRNA-1273 (Moderna)
Danthu, C.	France	Prospective cohort	159	74	61.1	64.8 ± 11.5	Transplant	BNT162b2 (Pfizer/BionTech)
Del Bello, A.	France	Retrospective cohort	396	396	65	59 ± 15	Transplant	BNT162b2 (Pfizer/BionTech)
Easdale, S.	UK	Retrospective cohort	55	55	61.8	50 [18–73]*	Transplant	n = 21 (BNT162b2 (Pfizer/BionTech)) or n = 34 (AstraZeneca ChAdOx1 nCoV-19 vaccine (AZ))
Ehmsen, S.	Denmark	Prospective cohort	524	524	NA	NA	Malignancy	(BNT162b2 (Pfizer/BionTech)) or (mRNA-1273 (Moderna))
Eliakim-Raz, N.	Israel	Prospective cohort	161	95	58	65 [56–72]*	Malignancy	BNT162b2 (Pfizer/BionTech)
Firket, L.	USA	Retrospective cohort	40	20	45	51.2	Transplant	BNT162b2 (Pfizer/BionTech)
Furer, V.	Israel	Prospective Cohort	807	686	30.7	59 [19–88]*	Autoimmune	BNT162b2 (Pfizer/BionTech)
Gavriatopoulou, M.	Greece	Prospective cohort	271	58	48.2	75 [63–81]*	Malignancy	BNT162b2 (Pfizer/BionTech) or AZD1222 vaccine (ASTRAZENECA/OXFORD)
Geisen, UM.	Germany	Retrospective cohort	68	42	35.7	50.5	Autoimmune	BNT162b2 (Pfizer/BionTech) or mRNA-1273 (Moderna)
Ghandili, S.	Germany	Retrospective cohort	82	82	59.8	67.5 [40–85] *	Malignancy	mRNA or AZD1222 (ASTRAZENECA/OXFORD)
Goshen-Lago, T.	Israel	Prospective cohort	493	232	57	66	Malignancy	BNT162b2 (Pfizer/BionTech)
Grupper, A.	Israel	Retrospective cohort	151	136	81.7	58.6	Transplant	BNT162b2 (Pfizer/BionTech)
Hagin, D.	Israel	Prospective cohort	26	26	42.4	48.4	Hereditary or Acquired immunodeficiency	BNT162b2 (Pfizer/BionTech)
Hall, V. G.	Canada	Prospective cohort	127	127	69.3	66.2 [63.4-70.6] *	Transplant	mRNA-1273 (Moderna)
Harrington, P.	UK	Retrospective cohort	21	21	33.3	52.4	Malignancy	BNT162b2 (Pfizer/BionTech)
Haskin, O.	Israel	Prospective cohort	52	38	66	18.6 ± 2.8	Transplant	BNT162b2 (Pfizer/BionTech)
Havlin, J.	Czech Republic	Prospective cohort	48	48	60.4	52.1 ± 14.3	Transplant	BNT162b2 (Pfizer/BionTech)
Herishanu, Y.	Israel	Prospective cohort	219	167	67.1	71 [63–76]*	Malignancy	BNT162b2 (Pfizer/BionTech)
Herrera, S.	Spain	Prospective cohort	104	104	79.8	60*	Transplant	mRNA-1273 (Moderna)
Herzog Tzarfati, K.	Israel	Prospective cohort	423	315	0.56	71 [61–78]*	Malignancy	BNT162b2 (Pfizer/BionTech)
Hod, T.	Israel	Prospective cohort	322	120	80	59.7 ± 13	Transplant	BNT162b2 (Pfizer/BionTech)
Holden, I.K.	Denmark	Prospective cohort	80	79	55	58.9 [47.9-66.8]*	Transplant	BNT162b2 (Pfizer/BionTech)
Iacono, D.	Italy	Cross-sectional	108	36	41.6	82*	Malignancy	BNT162b2 (Pfizer/BionTech)
Itzhaki Ben Zadok, O.	Israel	Prospective cohort	39	39	83	61 [44–69]*	Transplant	BNT162b2 (Pfizer/BionTech)
Karacin, C.	Turkey	Prospective cohort	47	47	61.7	73 [64–80]*	Malignancy	CoronaVac
Kennedy, NA.	UK	Prospective cohort	1293	1293	NA	NA	Autoimmune	n = 589 (BNT162b2 (Pfizer/BionTech)) or n = 704 )ChAdOx1 or AZD1222 (ASTRAZENECA/OXFORD))
Korth, J.	Germany	Prospective cohort	46	23	48	57.7	Transplant	BNT162b2 (Pfizer/BionTech)
Malard, F.	France	Retrospective cohort	225	195	60	68.9*	Malignancy	BNT162b2 (Pfizer/BionTech)
Marinaki, S.	Greece	Prospective cohort	150	34	79.4	60 [49.1-68.4]*	Transplant	BNT162b2 (Pfizer/BionTech)
Massarweh, A.	Israel	Prospective cohort	180	102	57	66 [56–72]*	Malignancy	BNT162b2 (Pfizer/BionTech)
Mazzola, A.	France	Retrospective cohort	168	143	71.3	61 [55–67]*	Transplant	BNT162b2 (Pfizer/BionTech)
Medeiros-Ribeiro, A. C.	Brazil	Prospective cohort	1092	910	23.1	51 [40–60]*	Autoimmune	CoronaVac
Monin, L.	UK	Prospective cohort	205	151	52	73 [64.5-79.5]*	Malignancy	BNT162b2 (Pfizer/BionTech)
Narasimhan, M.	USA	Retrospective cohort	73	73	74	65 [53.5-69.5]*	Transplant	n = 48 (BNT162b2 (Pfizer/BionTech)) or n = mRNA-1273 (Moderna)
Noble, J.	France	Prospective cohort	57	57	68.5	62 ± 13	Transplant	mRNA-1273 (Moderna)
Ou, M. T.	USA	Prospective cohort	609	585	40	58 [45–68]*	Transplant	BNT162b2 (Pfizer/BionTech)
Palich, R.	France	Retrospective cohort	135	110	40	66 [54–74]*	Malignancy	BNT162b2 (Pfizer/BionTech)
Peled, Y.	Israel	Prospective cohort	77	77	64	62 [49–68]*	Transplant	BNT162b2 (Pfizer/BionTech)
Pimpinelli, F.	Italy	Prospective cohort	128	92	53.2	70 [28–80]*	Malignancy	BNT162b2 (Pfizer/BionTech)
Prendecki, M.	UK	Prospective cohort	119	119	52.1	52 [39.9-63.9]*	Autoimmune	n = 85 (BNT162b2 (Pfizer/BionTech)) or n = 34 (ChAdOx1 or AZD1222 (ASTRAZENECA/OXFORD))
Rabinowich, L.	Israel	Cross-sectional	105	80	70	60.1	Transplant	BNT162b2 (Pfizer/BionTech)
Rashidi-Alavijeh, J.	Germany	Prospective cohort	63	43	60.5	57 [49–64]*	Transplant	BNT162b2 (Pfizer/BionTech)
Reuken, P.	Germany	Prospective cohort	55	28	53.6	42 [36–59]*	Hereditary or Acquired immunodeficiency	BNT162b2 (Pfizer/BionTech)
Rincon-Arevalo, H.	Germany	Prospective cohort	75	40	70	62.4 [51.25-69.5]*	Transplant	BNT162b2 (Pfizer/BionTech)
Rozen-Zvi, B.	Israel	Prospective cohort	308	308	64	57.5 ± 13.8	Malignancy	BNT162b2 (Pfizer/BionTech)
Ruddy, J. A.	USA	Prospective cohort	404	404	4	44 [36–57]*	Autoimmune	BNT162b2 (Pfizer/BionTech)
Sattler, A.	Germany	Prospective cohort	78	39	71.8	57.3	Transplant	BNT162b2 (Pfizer/BionTech)
Schramm, R.	Germany	Prospective cohort	100	50	64	55 ± 10	Transplant	BNT162b2 (Pfizer/BionTech)
Seyahi, E.	Turkey	Cross-sectional	382	82	35.4	42.2 ± 10	Autoimmune	BBIBP-CorV (Sinopharm)
Strauss, A.	USA	Prospective cohort	161	161	43	64 [48–69]*	Transplant	BNT162b2 (Pfizer/BionTech) or mRNA-1273 (Moderna)
Stumpf, J.	Germany	Prospective cohort	3100	368	65.5	57.3 ± 13.7	Transplant	n = 103 (BNT162b2 (Pfizer/BionTech)) or n = 265 (mRNA-1273 (Moderna))
Stumpf, J.	Germany	Prospective cohort	71	48	63	57±14.4	Transplant	BNT162b2 (Pfizer/BionTech)
Terpos, E.	Greece	Prospective cohort	152	48	60.4	83*	Malignancy	BNT162b2 (Pfizer/BionTech)
Terpos, E.	Greece	Prospective cohort	59	59	61	66 [61–76]*	Malignancy	BNT162b2 or AZD1222
Terpos, E.	Greece	Prospective cohort	502	276	54.7	74 [62–80]*	Malignancy	BNT162b2 or AZD1222
Thakkar, A.	USA	Retrospective cohort	200	200	42	67 [27–90]*	Malignancy	n = 180 (mRNA vaccines) or n = 20 (AD26.COV2.S)
Werbel, WA.	USA	Retrospective cohort	30	30	43.3	57 [44–62]*	Transplant	n = 17 (BNT162b2 (Pfizer/BionTech)) or n = 13 (mRNA-1273 (Moderna))
Yanay, NB.	Israel	Retrospective cohort	204	204	63.8	57.7 [49.4-67.5]*	Transplant	BNT162b2 (Pfizer/BionTech) or mRNA-1273 (Moderna)
Yi, SG.	USA	Prospective cohort	176	145	NA	NA	Transplant	BNT162b2 (Pfizer/BionTech) or mRNA-1273 (Moderna)

*reported values are median [interquartile range (IQR)]; otherwise are mean ± standard deviation (SD)

Quality assessment of the included studies is presented in Additional file 1: Table S1. The majority of the studies (n = 64) were of good quality and 16 had fair quality.

### Meta-analysis

#### First dose

**Fig. 2:**
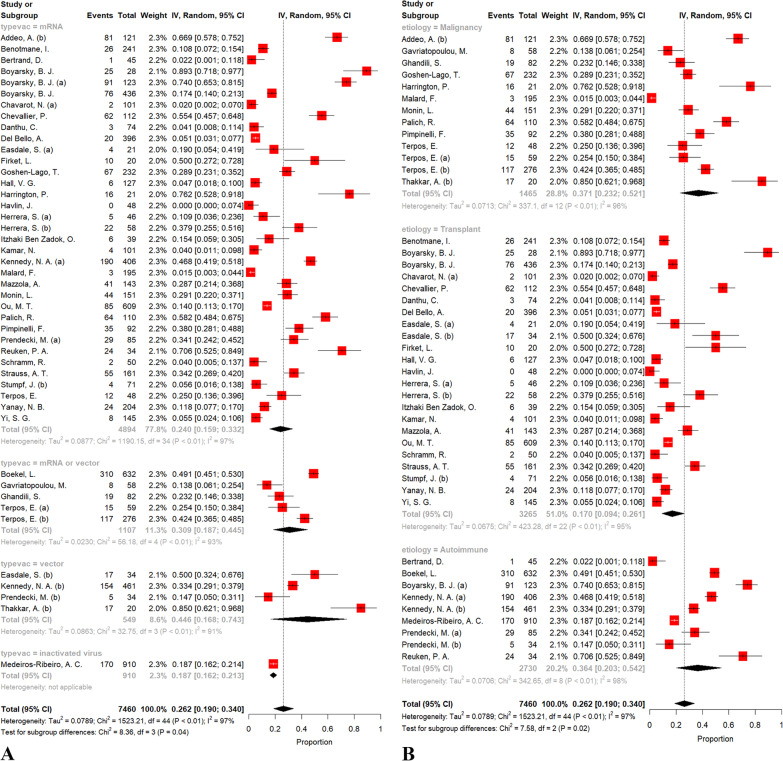
Forest plot of seroconversion proportions (prevalence) regarding the type of vaccine (**A**) and etiology of immunodeficiency (**B**) following the first dose of vaccine

**Fig. 3:**
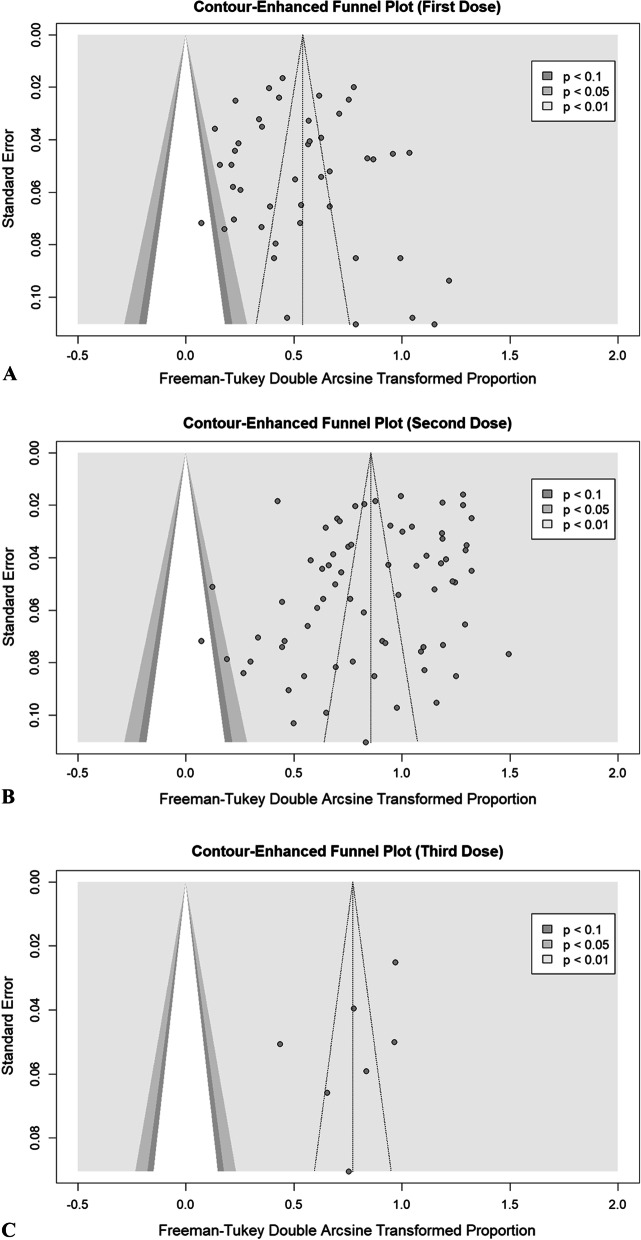
Counter-enhanced funnel plots regarding the publication bias following the first dose (**A**), second dose (**B**), and third dose (**C**) of vaccination.

Results of overall efficacy and between-group meta-analyses following the first, second, and third doses are presented in Table 2. The crude overall prevalence of seroconversion after the first dose administration in the pooled sample of 7460 individuals was 26.17% (95% CI: 19.01%; 33.99%, test of heterogeneity: I^2^ = 97.1%, *p *< 0.0001). Considering the type of vaccine, the test for subgroup differences showed significant results (*p* = 0.04, Fig. 2A). To investigate more, we conducted a pair-wised analysis to find whether there is a significant difference between mRNA and vector group. Accordingly, no significant difference was observed (*p* = 0.17). In addition, a pair-wised meta-analysis of combined group of mRNA and vector vaccines compared to inactivated group demonstrated a significant difference (30% vs. 18%, respectively; *p* = 0.04). Regarding the etiology, our primary analysis demonstrated a significant between-group difference (*p* = 0.02, Fig. 2B). Moreover, pair-wised analysis showed that the difference between malignancy and autoimmune group was not statistically significant (*p* = 0.95); however, malignancy vs. transplant (37% vs. 17%, *p* = 0.01) and autoimmune vs. transplant (36% vs. 17%, *p* = 0.04) exhibited statistically significant differences. Eggers' test does not indicate the presence of funnel plot asymmetry (*p* = 0.68); thus, the funnel plot implied no publication bias (Fig. 3A). There were no significant changes in the pooled prevalence or heterogeneity after eliminating each study in the sensitivity analysis (leave-one-out analysis) (Additional file 1: Fig. S1). As a result, none of the studies were able to explain the observed heterogeneity of results.

**Table 2:** Results of between-group meta-analyses

Vaccination dose	Sub-group	Comparison	No. studies	No. observations	No. events	Meta-analysis			Heterogeneity	
						Effect size (%)	95% Confidence interval (%)	*P value*	I^2^ (%)	*P* value
First dose	Overall		45	7460	1979	26.17	19.01, 33.99	–	97.1	< 0.0001
	Type of vaccine	mRNA	35	4894	1147	24.02	15.87, 33.2	0.0392	97.1	–
		mRNA or vector	4	1107	469	30.88	18.27, 44.53		92.9	
		Vector	5	549	193	44.59	16.8, 74.26		90.8	
		Inactivated	1	910	170	18.68	16.21, 21.28		–	
	Type of vaccine pair-wised	mRNA versus vector	39	5443	1340	25.89	17.82, 34.85	0.1790	97	< 0.0001
	Etiology	Malignancy	13	1465	498	37.05	23.19, 52.05	0.0226	96.4	–
		Transplant	23	3265	507	17.01	9.44, 26.15		94.8	
		Autoimmune	9	2730	974	36.4	20.35, 54.15		97.7	
	Etiology pair-wised	Malignancy versus autoimmune	22	4195	1472	36.76	26.3, 47.88	0.9514	96.9	< 0.0001
		Malignancy versus transplant	36	4730	1005	23.73	16.07, 32.32	0.0171	96.3	< 0.0001
		Autoimmune versus transplant	32	5995	1481	22.07	14.34, 30.88	0.0404	97.3	< 0.0001
Second dose	Overall		70	13181	8326	57.11	49.22, 64.83	–	98.4	< 0.0001
	Type of vaccine	mRNA	63	10441	6651	56.41	48.01, 64.64	< 0.0001	98.1	–
		mRNA or vector	2	908	777	82.83	58.24, 97.77		98.3	
		Vector	2	771	134	19.12	11.27, 28.37		49.7	
		Inactivated	3	1061	764	75.8	58.81, 89.46		91.3	
	Type of vaccine pair-wised	mRNA versus vector	65	11212	6785	55.28	46.98, 63.44	< 0.0001	98.4	< 0.0001
	Etiology	Malignancy	18	2879	2076	72.15	59.24, 83.45	< 0.0001	97.6	–
		Transplant	36	5836	2493	38.29	29.93, 46.99		96.2	
		Autoimmune	15	4440	3737	80.25	68.08, 90.14		96.7	
	Etiology pair-wised	Malignancy versus autoimmune	33	7319	5815	75.9	67.07, 83.76	0.3471	97.5	< 0.0001
		Malignancy versus transplant	54	8715	4571	49.93	41.36, 58.51	< 0.0001	97.8	< 0.0001
		Autoimmune versus transplant	51	10276	6230	51.21	41.94, 60.44	< 0.0001	98.6	< 0.0001
Third dose	Overall		7	909	505	48.65	36.43, 62.79	–	94.4	< 0.0001

mRNA, messenger ribonucleic acid

#### Second dose

**Fig. 4:**
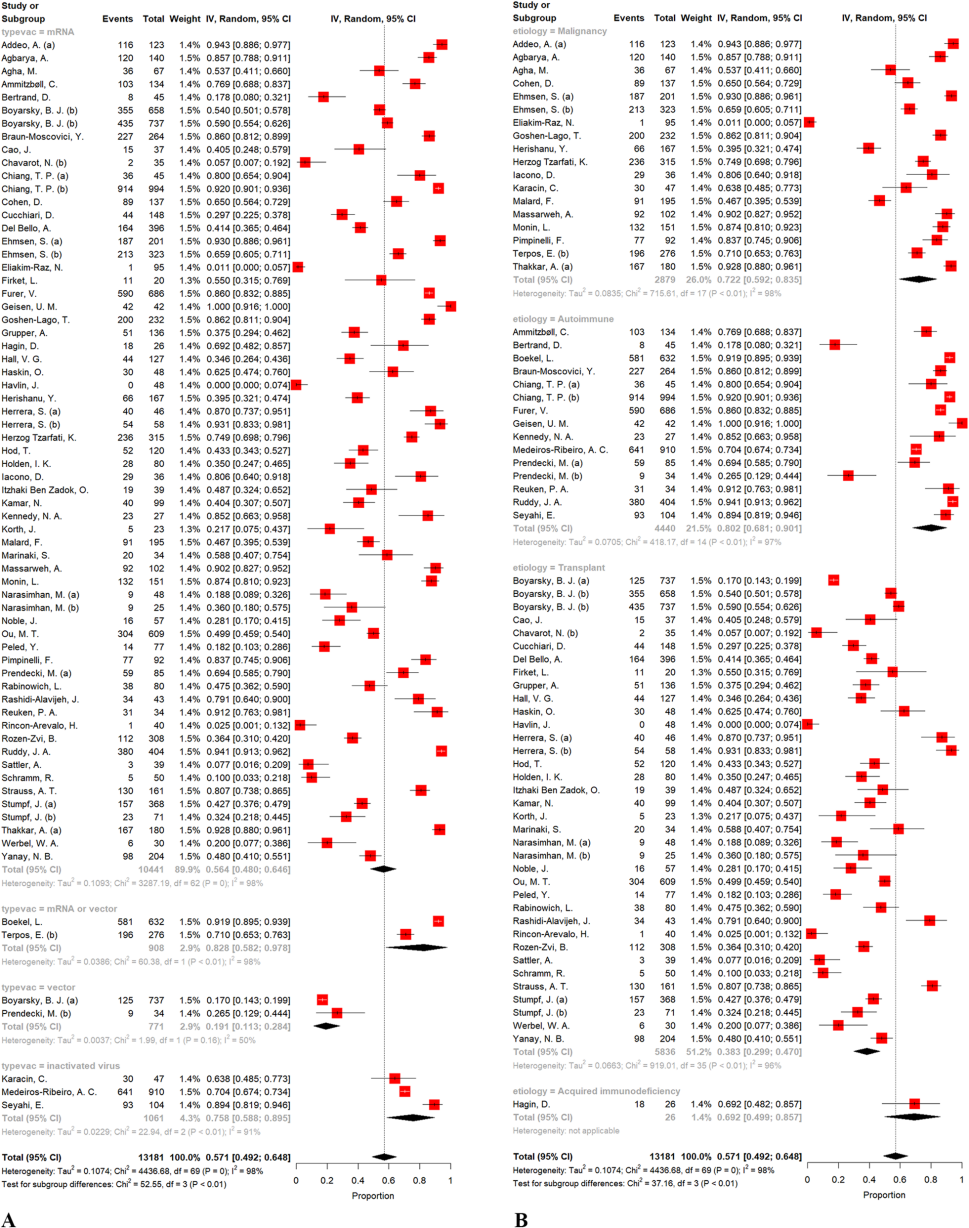
Forest plot of seroconversion proportions (prevalence) regarding the type of vaccine (**A**) and etiology of immunodeficiency (**B**) following the second dose of vaccine

Overall seroconversion prevalence following the second dosage in the pooled sample of 13,181 patients was 57.11% (95% CI: 49.22%; 64.83%, test of heterogeneity: I^2^ = 98.4%, *p* < 0.01). Given the vaccine's type, the test for subgroup differences yielded significant findings (*p * < 0.01, Fig. 4A). We performed a pair-wised analysis to see if the mRNA and vector groups differed significantly. As a result, a large disparity was discovered (*p *< 0.0001), mainly due to various patient recruitment methods. Furthermore, a significant difference was found in a pair-wised meta-analysis comparing the combined group of mRNA and vector vaccines to the inactivated group (83% vs. 76%, respectively; *p* = 0.04). A substantial between-groups difference was found with regards to the etiology (*p *< 0.01, Fig 4B). In addition, a pair-wise comparison of malignancy vs. transplant (72% vs. 38%, *p * < 0.001) and autoimmune vs. transplant (80% vs. 38%, *p *< 0.0001) groups found statistically significant differences between the analyzed groups; however, malignancy vs. autoimmune did not show any significant difference (72% vs. 80%, *p* = 0.34). Using Eggers' test, there was no evidence of asymmetry in the funnel plot (*p* = 0.06), suggesting no publication bias (Fig. 3B). After excluding each study in the sensitivity analysis (leave-one-out analysis), the aggregated prevalence and heterogeneity did not change (Additional file 1: Fig S2). For this reason, no one study could account for this wide range of outcomes.

Notably, considering immunocompromised patients due to autoimmune diseases on anti-TNF treatment, the seroconversion prevalence was estimated as 86.07% (95% CI: 63.16%; 99.23%, test of heterogeneity: I^2^ = 99.1%, *p* < 0.01).

#### Third dose

**Fig. 5:**
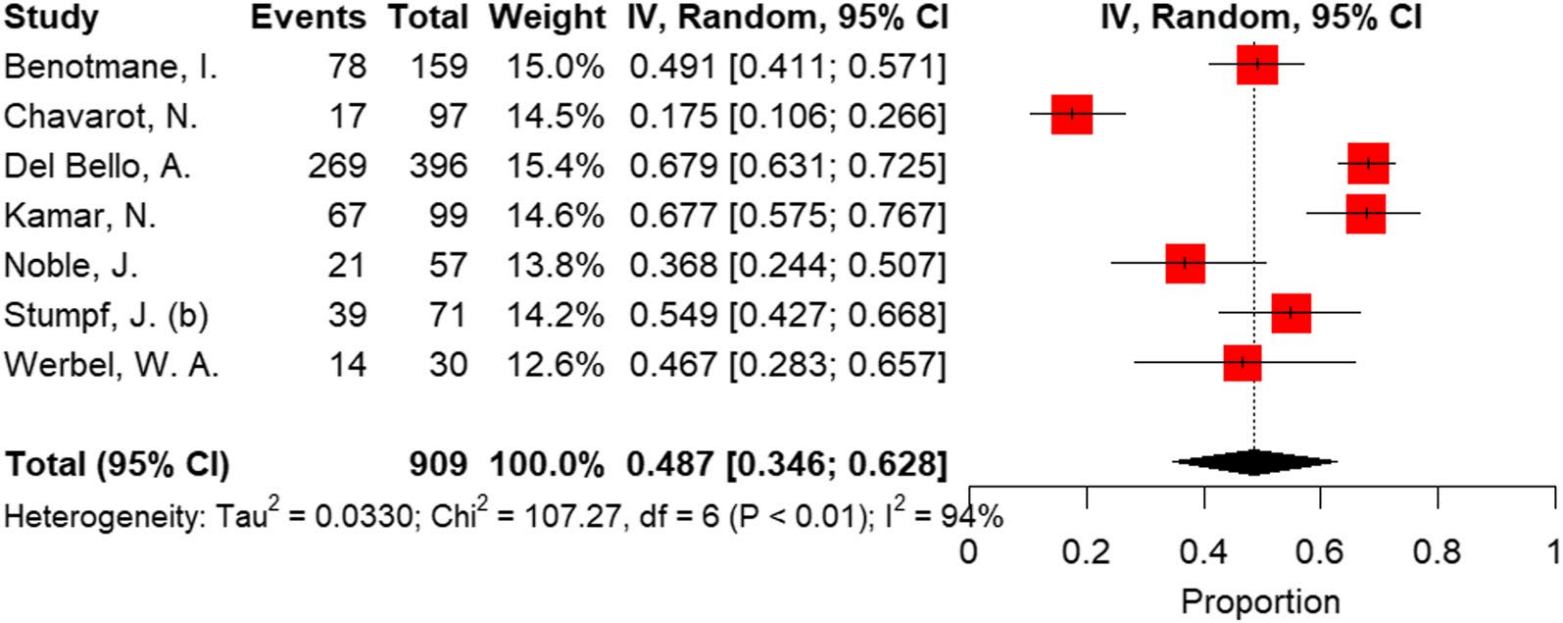
Forest plot of seroconversion proportions (prevalence) following the third dose of vaccine

All the included original studies in this analysis measured seroconversion after three doses of mRNA vaccines in transplant recipients. Overall prevalence of seroconversion in the combined sample of 909 transplant patients following the third dose of vaccine was 48.65% (95% CI: 34.63%; 62.79%, test of heterogeneity: I^2^ = 94.4%, *p * < 0.0001, Fig 5). Eggers' test revealed no indication of funnel plot asymmetry (*p* = 0.18), confirming that there was no publication bias (Fig. 3C). The pooled prevalence and heterogeneity remained unchanged after the sensitivity analysis (leave-one-out analysis) when each study was excluded (Additional file 1: Fig. S3). Thus, no single study could explain the heterogeneity of outcomes.

## Discussion

The pooled findings demonstrated a growing pattern of seroconversion rate after the administration of the second dose of COVID-19 vaccine compared to the first dose regardless of either vaccine type or the etiology of immunosuppression. Our findings also revealed a better response to mRNA vaccines compared to vector vaccines reaching significance after the administration of the second dose. In addition, transplant patients responded less robust compared to other IC patients regardless of the number of doses. It is worth mentioning that all the studies included in the pooled analysis of third-dose booster evaluated transplant patients; nevertheless, the rising pattern of seroconversion was observed even in this group of patients compared to the findings from both the first and second doses.

Viral vectors are modified viruses utilized to deliver the immunogenic part of the target virus [103]. On the other hand, mRNA vaccines deploy mRNAs coding specific viral proteins to trigger an immune response [103]. mRNA and vector vaccines seem to induce immunity with different mechanisms in healthy controls. Induction of SARS-CoV-2–specific IgG and neutralizing antibodies seems to be more pronounced with mRNA priming, while cellular immunity (including both SARS-CoV-2–specific CD4 and CD8 T cell levels) tends to be induced more robustly after vector priming [104]. However, this difference has been less prominent in IC patients [104]. Although our findings revealed higher rates of seroconversion after the second dose of mRNA vaccines, antibody assessment might be insufficient to compare immune response, and cellular immunity should be assessed as well [104].

Data regarding inactivated vaccines are rare; however, our findings show a significant difference between inactivated vaccines and combined groups of mRNA and vector vaccines. A previous report has also implicated lower efficacy of inactivated vaccines compared to vector vaccines in terms of antibody level and neutralization in immunosuppressed patients with rheumatic diseases [105]. These findings should be interpreted with caution as more studies are needed to unravel the efficacy of inactivated vaccines.

Intriguingly, a lower seroconversion rate was observed in transplant patients compared to other IC patients, even though a rising response rate was observed after boosting in this group of patients. Generally, transplant patients receive drugs that interfere with T and B cell activation and proliferation, posing an obstacle in the way of antibody generation [106]. Conspicuously, boosting seems to raise an immune response in all IC patients according to our findings, the fact which was observed with previous vaccines such as influenza [107].

Although we showed an acceptable rate of seroconversion among patients using anti-TNF therapy, reports show a persistent reduction in the titers of anti-SARS-CoV-2 spike protein antibody with time in patients with inflammatory bowel disease (IBD) who are on anti-TNF treatments [108]. While anti-TNF therapies can mitigate detrimental outcomes in severe COVID-19 due to dampening of the systemic inflammatory response, the reduction of antibodies over time might necessitate considering booster doses in these patients [108, 109].

We should mention that our study has some limitations. There was a lack of data regarding HIV and other hereditary or acquired immunodeficiency disorders and also inactivated vaccines. Besides, we included studies with both prospective and retrospective designs, which may decrease the level of evidence.

## Conclusion

For the first time, this meta-analysis compared seroconversion rate after administering different types of COVID-19 vaccines in IC patients at different time points of vaccination. The rising pattern of seroconversion after boosting tends to be promising; however, more attention should be devoted to transplant patients who possess the lowest response rate.

## Supplementary Information

**Additional file 1. Figure S1**. Results of Sensitivity analysis (leave-one-out analysis) of the First Dose meta-analysis (I^2^ and effect size plot). **Figure S2**. Results of Sensitivity analysis (leave-one-out analysis) of the Second Dose meta-analysis (I^2^ and effect size plot). **Figure S3**. Results of Sensitivity analysis (leave-one-out analysis) of the Third Dose meta-analysis (I^2^ and effect size plot). **Table S1**. Quality assessment using NIH tool.

## Data Availability

The authors stated that all information provided in this article could be shared.
