# Supplementary material for: Seroconversion following the first, second, and third dose of SARS-CoV-2 vaccines in immunocompromised population: a systematic review and meta-analysis
Source: Virol J. 2022 Aug 8;19:132. doi: 10.1186/s12985-022-01858-3 (PMC9358061; doi:10.1186/s12985-022-01858-3)
Supplement: Supplementary file 1 — Additional file 1. Figure S1. Results of Sensitivity analysis (leave-one-out analysis) of the First Dose meta-analysis (I2 and effect size plot). Figure S2. Results of Sensitivity analysis (leave-one-out analysis) of the Second Dose meta-analysis (I2 and effect size plot). Figure S3. Results of Sensitivity analysis (leave-one-out analysis) of the Third Dose meta-analysis (I2 and effect size plot). Table S1. Quality assessment using NIH tool. [file 12985_2022_1858_MOESM1_ESM.docx]

**Table of content**

**Page 2 ………………………………………………………………………………….. Fig S1**

**Page 3 ………………………………………………………………………………….. Fig S2**

**Page 4 ………………………………………………………………………………….. Fig S3**

**Page 5 ………………………………………………………………………………….. Table S1**

**Supplementary Figure S1. Results of Sensitivity analysis (leave-one-out analysis) of the First Dose meta-analysis (I^2^ and effect size plot)**

**
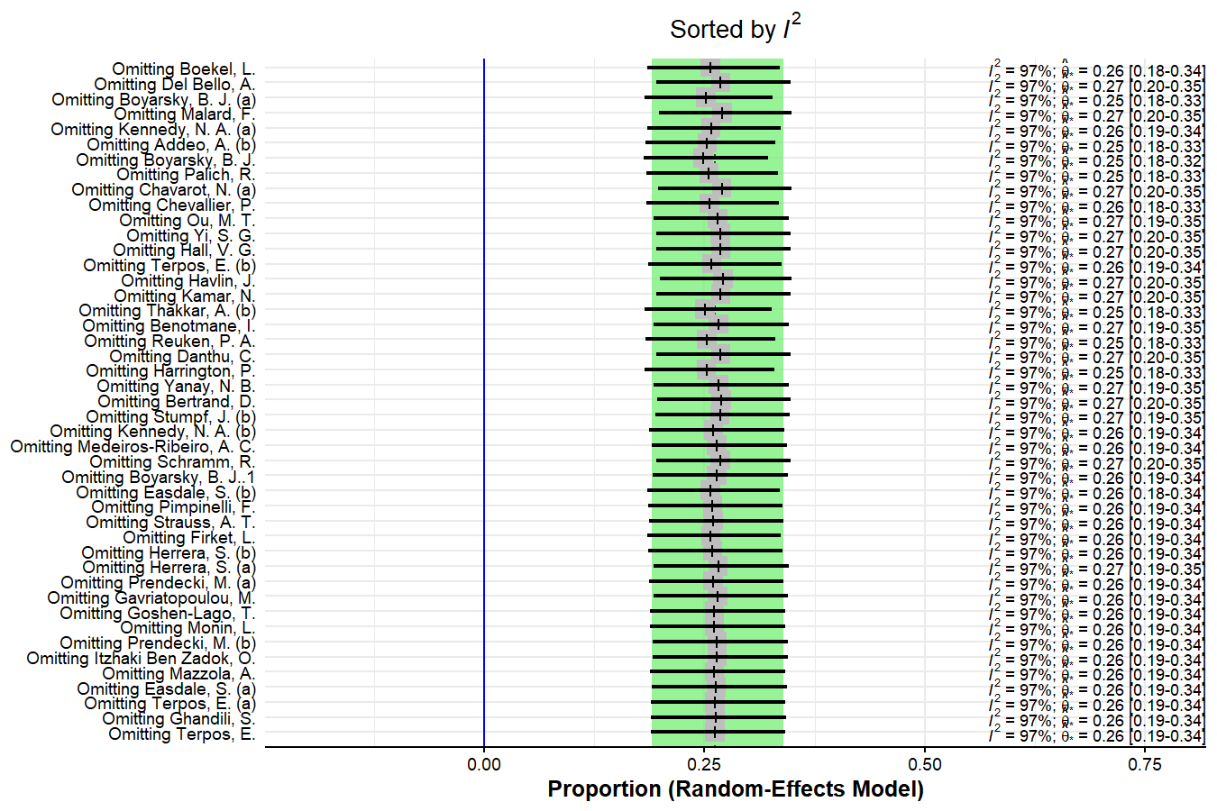

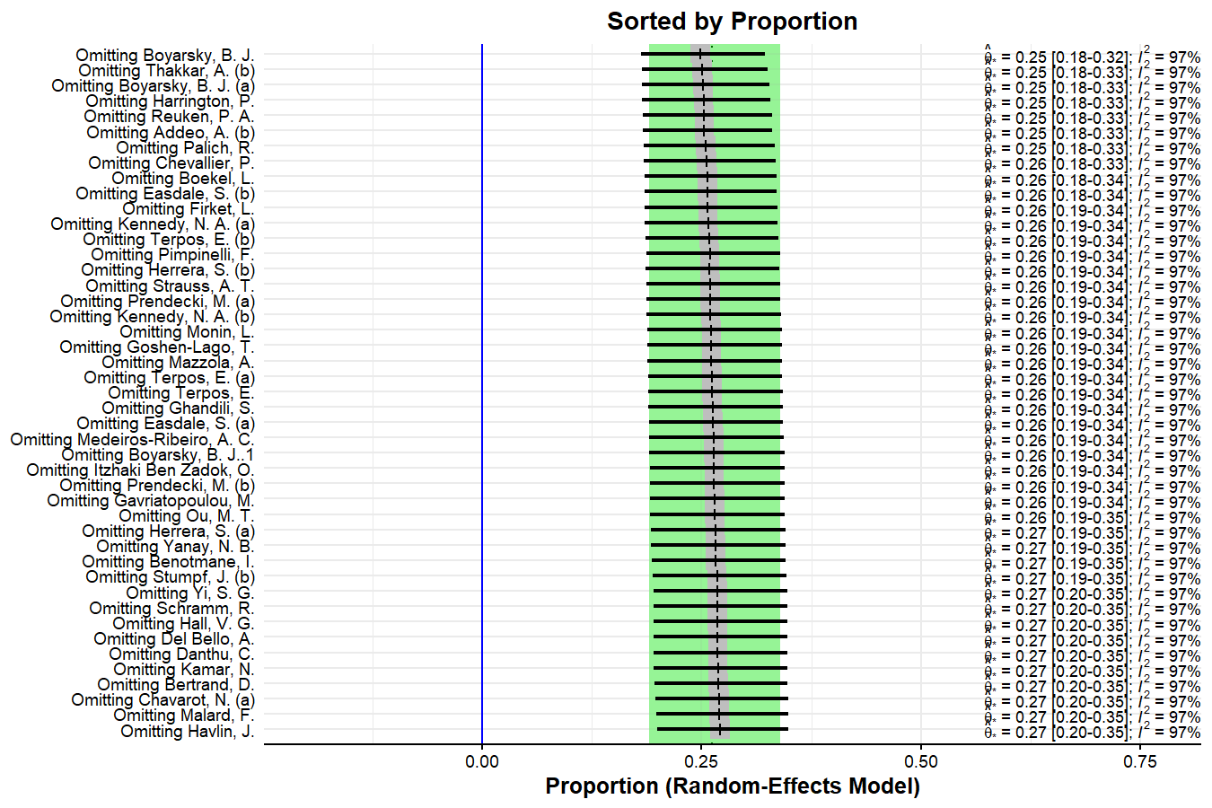
**

**Supplementary Figure S2. Results of Sensitivity analysis (leave-one-out analysis) of the Second Dose meta-analysis (I^2^ and effect size plot)**

**
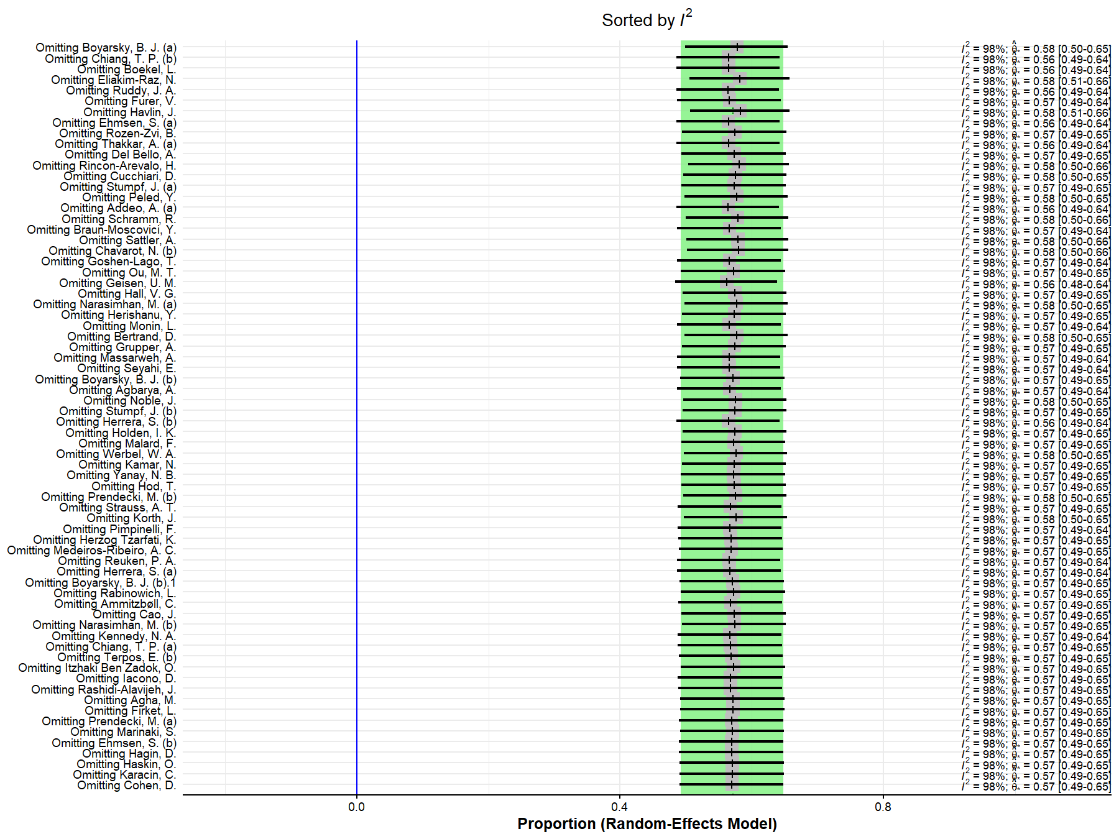

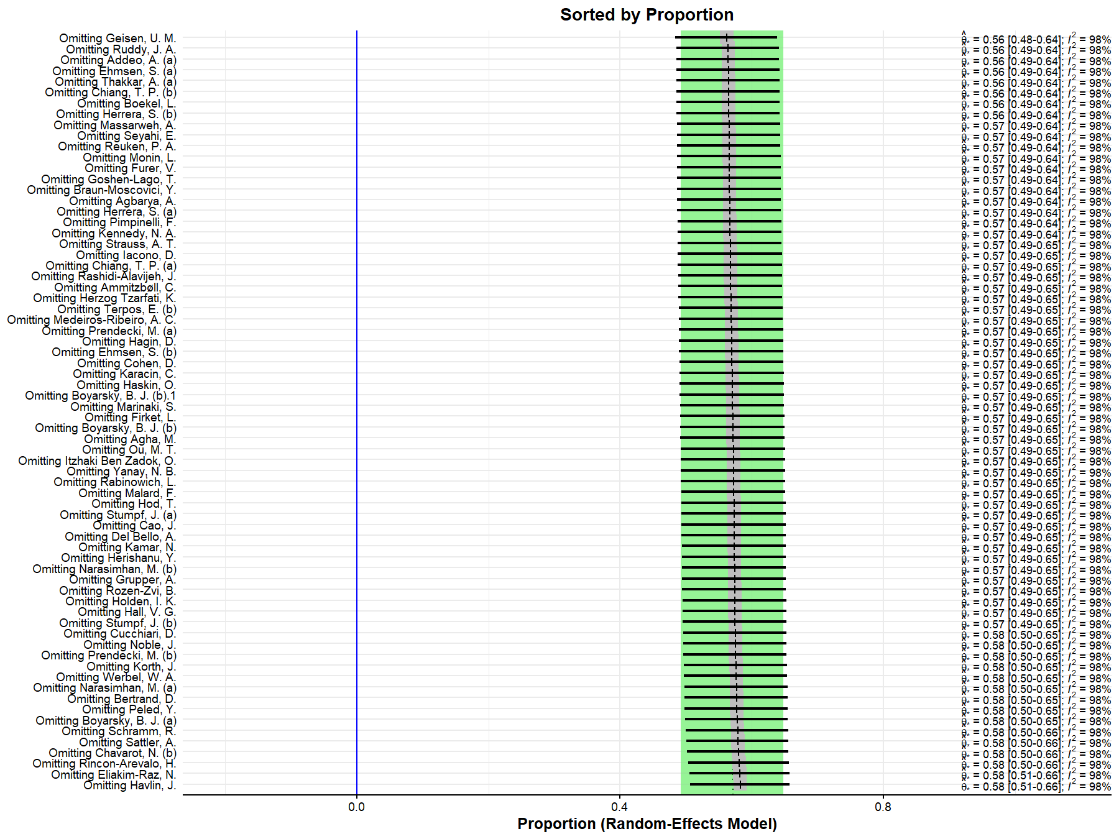
**

**Supplementary Figure S3. Results of Sensitivity analysis (leave-one-out analysis) of the Third Dose meta-analysis (I^2^ and effect size plot)**


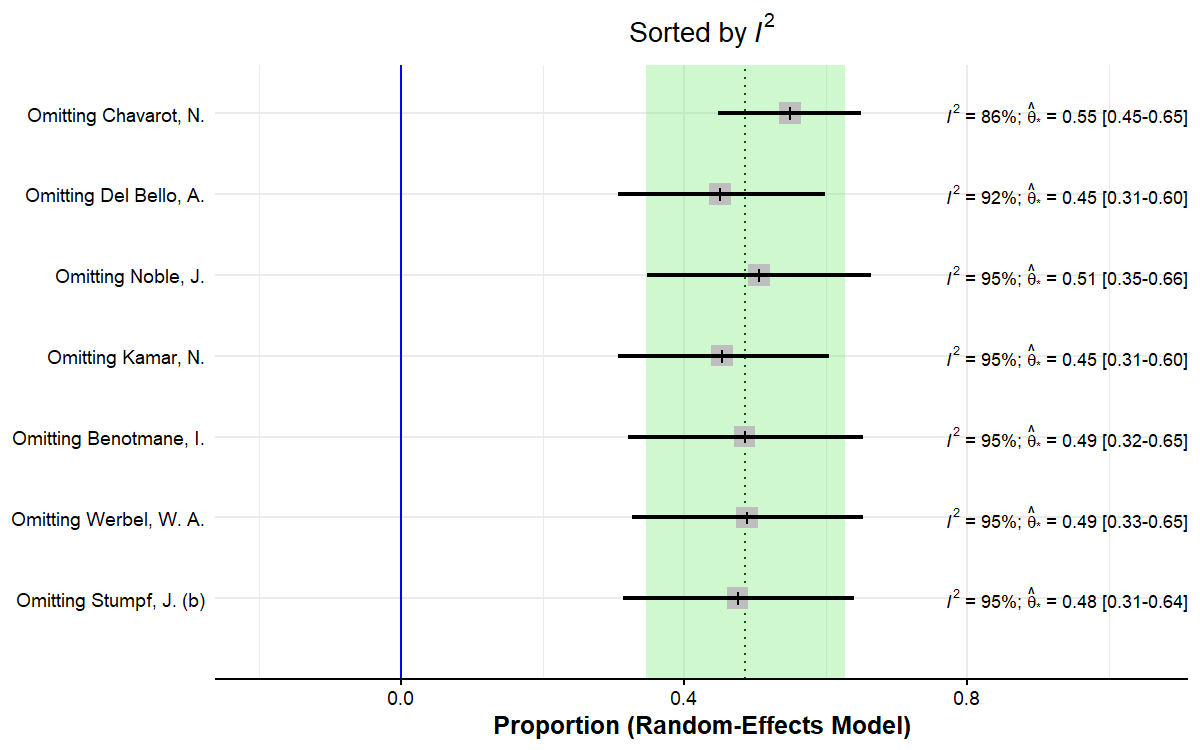

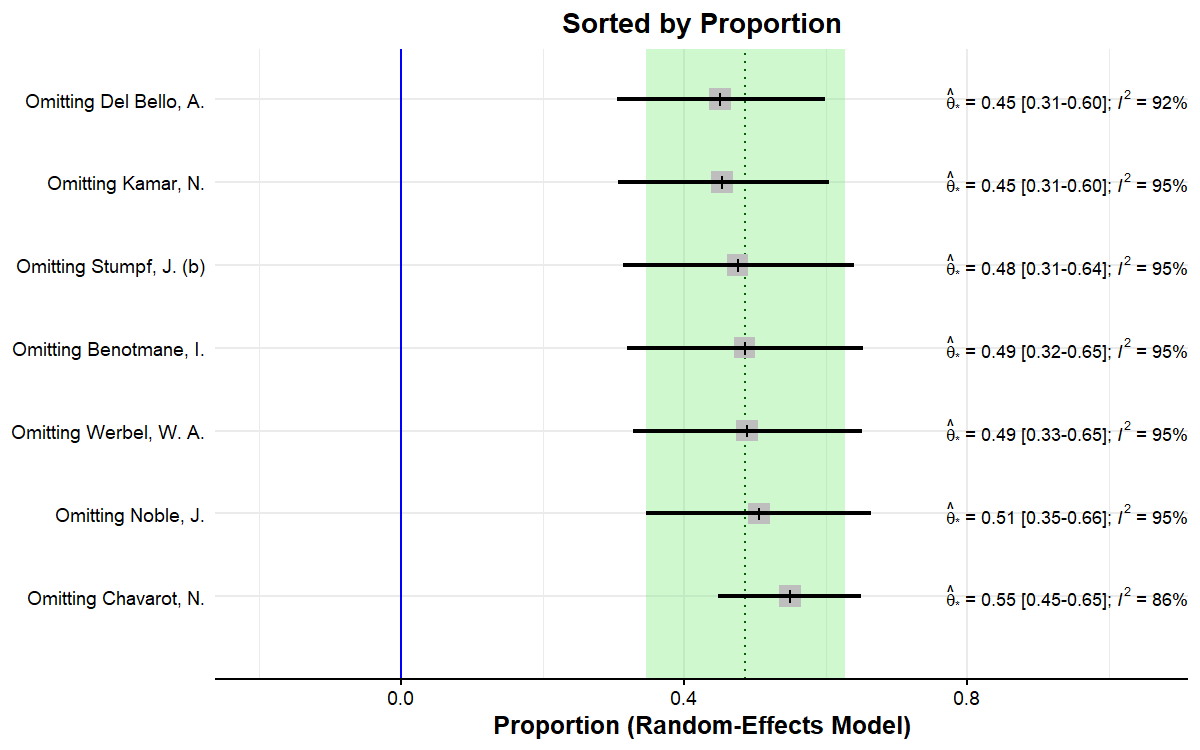


| **Table S1. Quality assessment using NIH tool** | | |
| --- | --- | --- |
| **With Control (Sum/14)** | | |
| **Study ID** | **Score** | **Quality Rating** |
| **Agbarya, A.** | 11 | Good |
| **Bertrand, D.** | 9 | Fair |
| **Boekel, L.** | 10 | Fair |
| **Boyarsky, B/** | 10 | Fair |
| **Braun-Moscovici, Y.** | 11 | Good |
| **Cao, J.** | 11 | Good |
| **Chavarot, N.** | 12 | Good |
| **Chevallier, P.** | 12 | Good |
| **Danthu, C.** | 11 | Good |
| **Eliakim-Raz, N.** | 11 | Good |
| **Firket, L.** | 11 | Good |
| **Furer, V.** | 10 | Fair |
| **Gavriatopoulou, M.** | 13 | Good |
| **Geisen, UM.** | 11 | Good |
| **Goshen-Lago, T.** | 11 | Good |
| **Grupper, A.** | 11 | Good |
| **Haskin, O.** | 11 | Good |
| **Herishanu, Y.** | 11 | Good |
| **Herzog Tzarfati, K.** | 12 | Good |
| **Hod, T.** | 12 | Good |
| **Iacono, D.** | 10 | Fair |
| **Korth, J.** | 13 | Good |
| **Malard, F.** | 11 | Good |
| **Marinaki, S.** | 9 | Fair |
| **Massarweh, A.** | 11 | Good |
| **Medeiros-Ribeiro, A. C.** | 12 | Good |
| **Monin, L.** | 11 | Good |
| **Narasimhan, M.** | 11 | Good |
| **Narasimhan, M.** | 12 | Good |
| **Palich, R.** | 12 | Good |
| **Peled, Y.** | 10 | Fair |
| **Pimpinelli, F.** | 12 | Good |
| **Prendecki, M.** | 11 | Good |
| **Rabinowich, L.** | 9 | Fair |
| **Rashidi-Alavijeh, J.** | 10 | Fair |
| **Reuken, P.** | 12 | Good |
| **Rincon-Arevalo, H.** | 14 | Good |
| **Sattler, A.** | 13 | Good |
| **Schramm, R.** | 11 | Good |
| **Seyahi, E.** | 11 | Good |
| **Stumpf, J.** | 12 | Good |
| **Terpos, E.** | 11 | Fair |
| **Terpos, E.** | 13 | Good |
| **Terpos, E.** | 10 | Good |
| **Yi, SG.** | 8 | Fair |
| **Without Control (Sum/12)** | | |
| **Study ID** | **Score** | **Quality Rating** |
| **Addeo, A.** | 11 | Good |
| **Agha, M.** | 10 | Good |
| **Ammitzbøll, C.** | 12 | Good |
| **Benotmane, I.** | 11 | Good |
| **Benotmane, I.** | 10 | Good |
| **Boyarsky, B. J.** | 8 | Fair |
| **Boyarsky, B. J.** | 10 | Good |
| **Boyarsky, B. J.** | 10 | Good |
| **Boyarsky, B. J.** | 11 | Good |
| **Chavarot, N.** | 8 | Fair |
| **Chiang, T. P.** | 9 | Good |
| **Cohen, D.** | 10 | Good |
| **Cucchiari, D.** | 10 | Good |
| **Del Bello, A.** | 8 | Fair |
| **Easdale, S.** | 10 | Good |
| **Ehmsen, S.** | 11 | Good |
| **Ghandili, S.** | 10 | Good |
| **Hagin, D.** | 9 | Good |
| **Hall, V. G.** | 11 | Good |
| **Harrington, P.** | 10 | Good |
| **Havlin, J.** | 11 | Good |
| **Herrera, S.** | 12 | Good |
| **Holden, I. K.** | 8 | Fair |
| **Itzhaki Ben Zadok, O.** | 9 | Good |
| **Karacin, C.** | 11 | Good |
| **Kennedy, NA.** | 9 | Good |
| **Noble, J.** | 10 | Good |
| **Ou, M. T.** | 11 | Good |
| **Rozen-Zvi, B.** | 10 | Good |
| **Ruddy, J. A.** | 11 | Good |
| **Strauss, A.** | 10 | Good |
| **Stumpf, J.** | 8 | Fair |
| **Thakkar, A.** | 10 | Good |
| **Werbel, WA.** | 10 | Good |
| **Yanay, NB.** | 10 | Good |
